# True rise in anaphylaxis incidence: Epidemiologic study based on a national health insurance database

**DOI:** 10.1097/MD.0000000000005750

**Published:** 2017-02-03

**Authors:** Min-Suk Yang, Ju-Young Kim, Byung-Keun Kim, Heung-Woo Park, Sang-Heon Cho, Kyung-Up Min, Hye-Ryun Kang

**Affiliations:** a Department of Internal medicine, SMG-SNU Boramae Medical Center; b Institute of Allergy and Clinical Immunology, Seoul National University Medical Research Center, Seoul; c Department of Internal Medicine, Gyeongsang National University Hospital, Jinju; d Department of Internal medicine, Seoul National University Bundang Hospital, Seongnam; e Department of Internal medicine, Seoul National University College of Medicine; f Drug Safety Monitoring Center, Seoul National University Hospital, Seoul, Korea.

**Keywords:** anaphylaxis, epidemiology, ICD-10, Korea, trends

## Abstract

The incidence trend of anaphylaxis in Asia is not well investigated. The aim of this study is to estimate the entire population-based incidence of anaphylaxis in Korea using a nationwide administrative database.

Data over a 7-year period (2008–2014) was obtained from the Korean National Health Insurance (NHI) claims database which covers 97.9% of the entire Korean population. Using diagnosis codes from the International Classification of Diseases-10 for anaphylaxis (T78.0, T78.2, T80.5, and T88.6), we identified the annual number of patients who had visited any hospital with a primary diagnosis of anaphylaxis. Incidence rates were calculated using the population distribution data of all NHI beneficiaries.

The incidence of anaphylaxis in Korea was 32.19 episodes per 100,000 person-years in 2014, which nearly doubled from 2008 (16.02 episodes per 100,000 person-years). The incidence of anaphylaxis increased continuously throughout these years regardless of gender and age groups (*P* for trend < 0.001). Female was significantly less predisposed than male (adjusted odds ratio [OR], 0.69; 95% confident interval [CI], 0.66–0.72; *P* < 0.001). The incidence was the lowest in 0 to 19 age group and the highest in 40 to 69 age group (adjusted OR, 2.41; 95% CI, 2.29–2.54; *P* < 0.001).

In conclusion, we report the increasing time trend of anaphylaxis incidence rates using nationwide claims database for the first time in Asia.

## 1 Introduction

Anaphylaxis is an acute-onset, potentially life-threatening, systemic allergic reaction.^[1]^ People of any age, even without past medical problems, can be afflicted by a near-fatal anaphylactic event. Despite its importance as a public health issue, precise estimates of the epidemiology of anaphylaxis are difficult to make because of the unexpected nature of this condition, previous lack of consensus on its diagnostic criteria, as well as the “underdiagnosis” by physicians.^[2]^ Previous studies on anaphylaxis epidemiology have reported an annual incidence rate of 1.5–50 per 100,000 person-years^[3,4]^, and there is evidence of an increasing trend.^[5–10]^ However, the majority of the reports are from Western countries such as the US,^[5,9]^ UK,^[6,7]^ and Australia^[8,10]^ and whether this rising trend applies to other global regions or not needs investigation.

Most of the reports on anaphylaxis epidemiology in Korea were conducted in special populations such as patients admitted to tertiary hospitals. A study from a tertiary hospital in Seoul, the capital city of Korea, reported 138 cases of anaphylaxis.^[11]^ The causes of anaphylaxis were drug (34.8%), food (21.0%), idiopathic (13.0%), exercise (13.0%), and insect sting (11.6%). Another study from a tertiary hospital in Suwon city found the causes of 158 anaphylaxis cases to be drug (51.2%), insect sting (25.3%), food (10.8%), and exercise (6.3%).^[12]^ Such discrepancy in the culprits of anaphylaxis is probably due to the small number of cases and regional differences. To overcome such a problem, a multicenter case study has been conducted in 15 university hospitals in Korea.^[13]^ The study used ICD-10 diagnostic codes to detect 1806 anaphylaxis patients between 2007 and 2011. A steady increase in the ratio of anaphylaxis cases to total number of patients was noted in all hospitals during the 5-year period. However, since the study provided only an indirect estimation of incidence confined to a very specific population, the data were insufficient to represent the true epidemiological trend of anaphylaxis in Korea.

For precise epidemiologic data, a longitudinal study based on a large standardized population is required, but these studies are costly as well as difficult to perform. In Korea, a nationwide database that includes the people of the entire country is available due to a single mandatory medical insurance system established by the government in 1989. Since the data of the entire nation is gathered by the same method every year, it is useful for evaluating serial trends of disease epidemiology. Here, we investigated the incidence of anaphylaxis using the nationwide claims database to obtain insight into the recent trends of anaphylaxis in Korea.

## 2 Methods

A retrospective, population-based study was performed including all patients who visited any medical institution in Korea using nationwide claims data from 2008 to 2014. This study used the Korean National Health Insurance (NHI) claims database. In Korea, the government runs a mandatory health insurance program covering all the citizens living in the country. The NHI program covered 49,989,620 beneficiaries in 2013, which represents 97.9% of the population in the Republic of Korea. All billing claims are submitted by health service providers to the Health Insurance Review & Assessment Service (HIRA), a government-affiliated agency, using the diagnostic and procedure codes of the International Classification of Diseases-10.^[14]^ HIRA provides open access to the recent nationwide data of NHI claims, which includes the annual number of inpatients and outpatients with specific disease codes (access at http://opendata.hira.or.kr/op/opc/olap3thDsInfo.do). We obtained cases from the NHI claims database for the period of 2008–2014 having a principal diagnosis of anaphylaxis with the following ICD-10 diagnostic codes: T78.0 (anaphylactic shock or reaction due to adverse food reaction); T78.2 (unspecified); T80.5 (anaphylactic reaction due to serum); T88.6 (due to adverse effect of correct drug or medicament properly administered).

The annual incidence rate of anaphylaxis was calculated by dividing the annual number of incident anaphylaxis cases by the total number of NHI beneficiaries for the corresponding year. Incidence rates were expressed as the number of cases per 100,000 person-years (PY). Data on the specific number of beneficiaries for each gender and age group were obtained from the “National Health Insurance Statistical Yearbook” published annually by HIRA.

Chi-square tests for trends were performed to verify whether there were the linear tendencies by years. To take a view of the changes in the incidence of anaphylaxis, incidence curves of each year, each gender, and each age groups were generated. As we found a few differences between the curves, a multivariate analysis with interaction variables that were constructed with age groups, gender, and year to confirm the differences in the shape of incidence curve was performed. To evaluate the risk factors for anaphylaxis, univariate and multivariate logistic regression analyses were used with year, gender, age group, and the interaction terms between the variables. All the analyses were done with IBM SPSS statistics version 20.0 (IBM).

## 3 Results

The number of all anaphylaxis episodes during 7-year study period (2008–2014) was 75,918. The subtypes of anaphylaxis were in the order of unspecified (T78.2, 83%), food (T78.0, 10%), drug (T88.6, 6.6%), and serum (T80.5, 0.4%) based on ICD codes.

**Figure 1:**
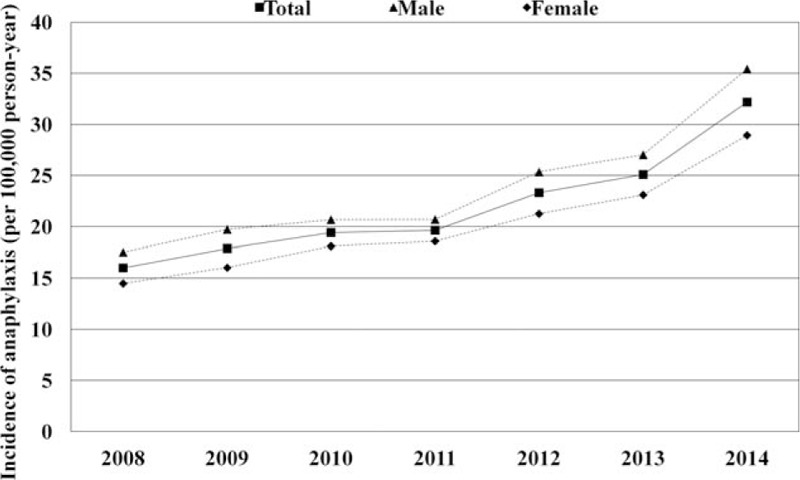
Annual incidence of anaphylaxis in Korea during the study period.

**Figure 2:**
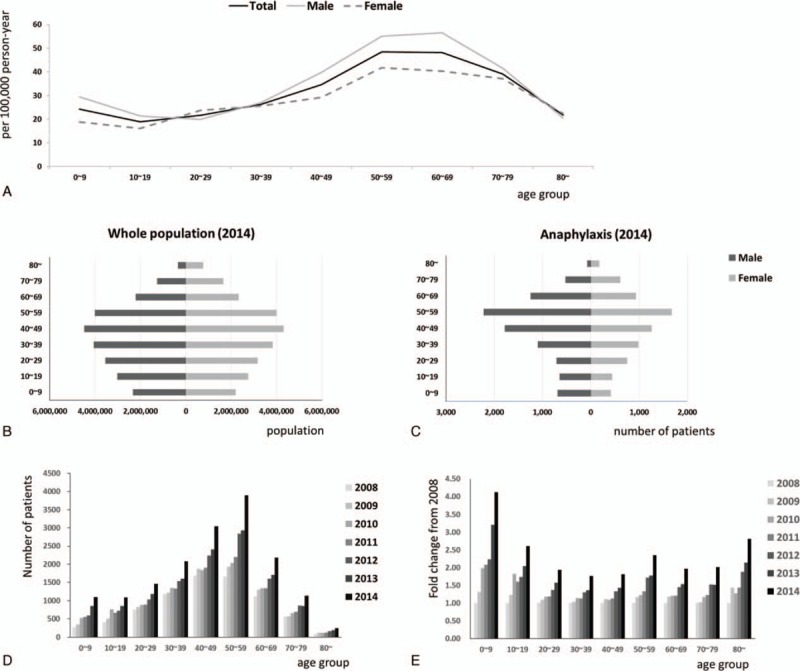
The incidence of anaphylaxis in Korea per age groups. The number of anaphylactic patients during study period (A), population structure (B), and the numbers of anaphylactic patients (C) in Korea in 2014, the gender differences in the incidence of anaphylaxis in 2014 (D) and the fold change of the anaphylactic patients during study period (E).

Mean annual incidence of anaphylaxis in Korea during study period was 22.01 per 100,000 person-years. The crude incidence rate of anaphylaxis was 32.19 per 100,000 person-years in 2014. According to gender, the incidence in males was consistently higher than that in females throughout the years (mean incidence 23.85 for male vs 20.06 for female per 100,000 person-years, *P* < 0.001) and also in the last year of the study period, or 2014 (the crude incidence rate 35.41 for male vs 28.93 for female, *P* < 0.001) (Fig. 1 and Table 1). The risk of anaphylaxis in female was significantly lower than in male (odds ratio [OR], 0.92; 95% confident interval [CI], 0.906–0.932; *P* < 0.001) in univariate analysis. Age groups were also related to the development of anaphylaxis. The number of anaphylaxis increased with age and peaked in the 50 to 59 age group (Fig. 2A), which showed remarkable rise after 4th decade allowing that the increase of middle-aged group (Fig. 2B and C). The incidence of anaphylaxis by age group was the lowest in teenagers (18.88 per 100,000 person-years in 2014), increased with age, peaked in the 6th decade (48.52 per 100,000 person-years in 2014), and decreased thereafter (Fig. 2A).

**Table 1:**
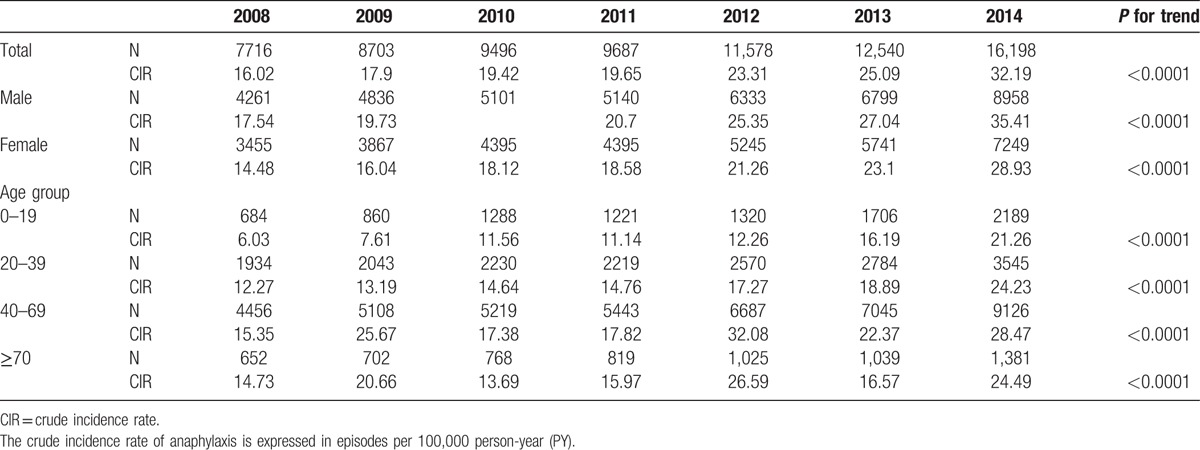
Crude incidence rate of anaphylaxis by sex and age groups.

There was a nearly 2-fold increase in the incidence rate (16.02 and 32.19 episodes per 100,000 person-years, respectively) as well as in the absolute numbers of episodes (7716 and 16,198 episodes, respectively) between 2008 and 2014. The incidence of anaphylaxis increased continuously throughout these years (Fig. 1) regardless of gender and age groups (*P* for trend < 0.001, Table 1). The risk of anaphylaxis increased by 1.11 (95% CI, 1.105–1.113; *P* < 0.001, Table 2) every year in univariate analysis during the study period. These increasing trends were observed in all age groups and most striking in 0 to 9 age group (Fig. 2D and E).

**Table 2:**
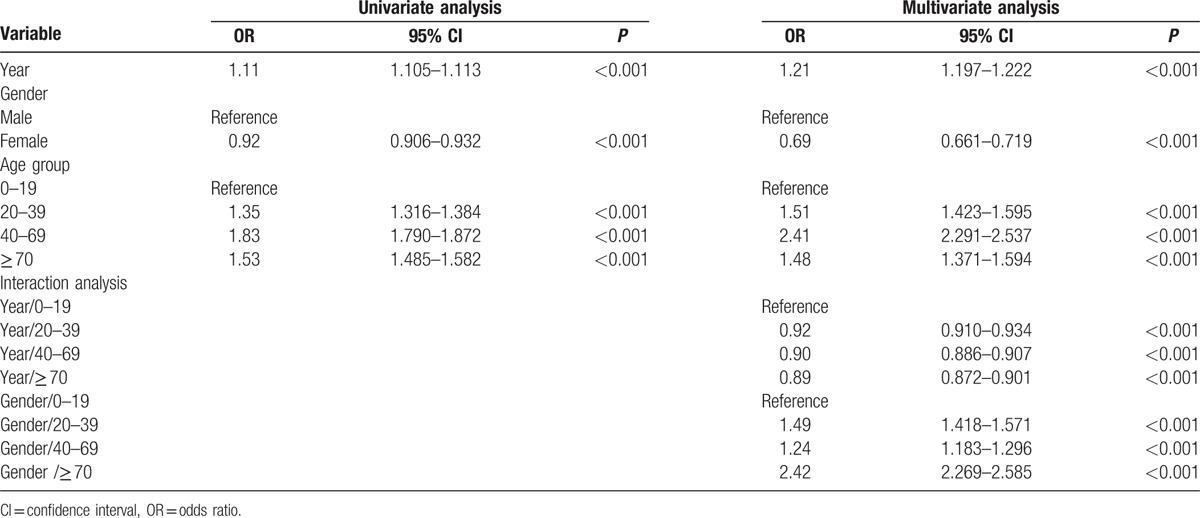
Factors associated with the incidence of anaphylaxis.

Since the interaction term with gender and year did not showed statistical significance (OR = 1.00; 95% CI, 0.993–1.008; *P* = 0.89), 3 main effects (year, gender, and age group) and 2 interactions (“year and age group” and “gender and age group”) were included in the final model. The influence of all 3 main effects on the incidence of anaphylaxis became bigger after multivariate analysis (Table 2). The influence of age on the incidence of anaphylaxis was the highest in the 40 to 69 age group (OR, 2.41; 95% CI, 2.291–2.537; *P* < 0.001) compared with other age groups (Table 2). The year-age group interaction in the 0 to 19 age group was significantly stronger than in other age groups (Table 2). The interactions between gender and age groups were significant in all age groups.

## 4 Discussion

In the present study, we investigated the incidence and its rising trend of anaphylaxis in Korea using nationwide claims data. A total of 75,918 patients had a primary diagnosis of anaphylaxis and the mean annual incidence of anaphylaxis was 22.01 per 100,000 person-years during the 7-year period. The incidence rate of anaphylaxis had grown nearly 2-fold during the study period (from 16.02 to 32.19 episodes per 100,000 person-years).

To our knowledge, this is the first study based on a nationwide claims database reporting the incidence trends of anaphylaxis in Asia. A rapid and continuous increase in the incidence of anaphylaxis was found during the study period. This trend had previously been reported repeatedly by many investigators from other countries. In the United Kingdom (UK), patient discharges from National Health Service hospitals with a diagnosis of anaphylaxis increased from 5.6 instances per 100,000 in 1991–1992 to 10.2 in 1994–1995 (*P* < 0.001).^[15]^ There was also a 5-fold increase in anaphylaxis admissions among patients younger than 20 years in New York, United States, from 1 to 4.6 per 100,000 person-years between 1990 and 2006.^[16]^ The anaphylaxis admission rates in Australia more than doubled from 3.6 to 8.0 per 100,000 person-years between 1994 and 2005 and such change occurred across all age groups.^[10]^ They showed that the rate of hospital admissions for anaphylaxis increased by 8.8% per year. The 0 to 4 age group showed the largest increase of hospital admission in this study from 4.1 to 19.7 per 100,000 population over the 12-year study period.^[10]^ In the present study, the incidence rate of anaphylaxis was 16.41 to 31.25 per person-years which was very higher than previous studies mentioned above.^[10,15,16]^ As there were differences in the design and timing of study, we could not directly compare our results from the others. However, there was a possibility that Asian population would be more susceptible to anaphylaxis than other ethnicities.

The overall incidence of anaphylaxis was higher in males than in females. Males were significantly more predisposed to anaphylaxis than females (OR, 1.21; 95% CI, 1.20–1.22; *P* < 0.001). The influence of gender in the incidence of anaphylaxis had been investigated in several studies. In Australia study mentioned above showed that among 0 to 14 age group, rates of admission for anaphylaxis were higher in boys than in girls, whereas in those aged 15 years and older, rates of admission were higher in female than in male. The gender difference seemed decreased in patients with 65 years and older.^[10]^ The male predominance in the younger age group was also reported from New York study that the rate of admission for anaphylaxis among subject younger than 20 years was significantly greater in male than in female (risk ratio, 1.45; 95% CI, 1.26 –1.66).^[16]^ However, in the UK study, the gender difference in the incidence of anaphylaxis was reversed from our study that the incidence of anaphylaxis in the UK was higher in women with a rate ratio of 1.20 (95% CI 1.11–1.31) which was largely attributed to females aged between 35 and 55 years.^[15]^

Age-specific incidence rates showed a left-skewed shape. The incidence of anaphylaxis was highest in the 40 to 69 years age group. In the UK study, crude incidence rate increased during childhood and peaked in adults between 15 and 55 years,^[15]^ whereas a study from Australia showed the highest incidence of anaphylaxis in children between 0–14 years^[17]^ and a study from the United States showed the highest incidence in the 70 to 79 years age group.^[18]^ Such differences in age-specific incidence rates may be due to the differences in the cause of anaphylaxis among countries. Age-specific differences in the cause of anaphylaxis were evaluated in previous report from the UK and food was predominant cause of anaphylaxis among children and young adults in contrast drug was the biggest cause among elderlies.^[19]^ However, we could not define the specific causes of anaphylaxis in this study because most of the cases were coded as unspecified anaphylaxis.

This study has several limitations. In the results of the multicenter case study from Korea, drug (46.5%) and food (24.2%) induced anaphylaxis were the most frequent followed by insect stings (16.4%), exercise (5.9%), and unknown etiology (7.0%).^[13]^ However, we could not evaluate the incidence of each subtype of the anaphylaxis in this study since most of the anaphylaxis cases were coded as classified as T78.2 (unspecified). Especially insect sting was reported as one of the major causes of anaphylaxis in previous reports of United States but insect sting-related anaphylaxis was unable to extract because of unavailability of compatible ICD code. The inability to include insect sting anaphylaxis is a common problem with studies using ICD-10 codes to detect anaphylaxis. There may be a problem with the accuracy of diagnostic coding. In some instances, alternative diagnostic codes such as acute urticaria, angioedema, and asthma attack might be used in real-life practice rather than anaphylaxis. To overcome such problem, using an appropriate operational definition (e.g., combining epinephrine prescription data) to detect anaphylaxis cases would be needed.

In conclusion, we report the increasing time trend of anaphylaxis incidence rates using nationwide claims database for the first time in Asia.
